# Sentiment Analysis of Social Media Data on Ebola Outbreak Using Deep Learning Classifiers

**DOI:** 10.3390/life14060708

**Published:** 2024-05-30

**Authors:** Alex Mirugwe, Clare Ashaba, Alice Namale, Evelyn Akello, Edward Bichetero, Edgar Kansiime, Juwa Nyirenda

**Affiliations:** 1 School of Public Health, Makerere University, Kampala P.O. Box 7072, Uganda; 2 Department of Statistical Science, University of Cape Town, Cape Town 7700, South Africa

**Keywords:** Ebola, deep learning, sentiment analysis, natural language processing

## Abstract

The Ebola virus disease (EVD) is an extremely contagious and fatal illness caused by the Ebola virus. Recently, Uganda witnessed an outbreak of EVD, which generated much attention on various social media platforms. To ensure effective communication and implementation of targeted health interventions, it is crucial for stakeholders to comprehend the sentiments expressed in the posts and discussions on these online platforms. In this study, we used deep learning techniques to analyse the sentiments expressed in Ebola-related tweets during the outbreak. We explored the application of three deep learning techniques to classify the sentiments in 8395 tweets as positive, neutral, or negative. The techniques examined included a 6-layer convolutional neural network (CNN), a 6-layer long short-term memory model (LSTM), and an 8-layer Bidirectional Encoder Representations from Transformers (BERT) model. The study found that the BERT model outperformed both the CNN and LSTM-based models across all the evaluation metrics, achieving a remarkable classification accuracy of 95%. These findings confirm the reported effectiveness of Transformer-based architectures in tasks related to natural language processing, such as sentiment analysis.

## 1. Introduction

The Ebola virus disease (EVD), also called Ebola haemorrhagic fever, is a highly contagious and deadly disease that is caused by four viruses within the genus Ebolavirus (EBOV) [1]. The four viruses are *Sudan virus* (SUDV), *Taï Forest virus* (TAFV), *Bundibugyo virus* (BDBV), and *Ebola virus* (EBOV) [1]. The EBOV was first discovered in humans during the South Sudan and Democratic Republic of Congo (DRC) outbreaks of 1976 [2,3], and since then, several outbreaks have been registered in different parts of the world, especially in Africa as follows: DRC in 1995, 2002, and between 2018 and 2019; Uganda in 2000, 2007, and 2022; and West Africa between 2014 and 2016. EVD outbreaks usually start from a single case of probable zoonotic transmission, followed by human-to-human transmission through contact with infected body fluids or corpses [4]. The virus causes severe and acute systemic disease with a high mortality rate. The 1995 outbreak in the DRC killed 245 out of the 317 people who were infected (a case fatality rate of 77.3%) [5], whilst the West Africa outbreak of 2014 had a fatality rate of 52% in Sierra Leone [6] and a fatality rate of over 50% in each of Guinea, Liberia, and Nigeria [7]. Overall, a total of 35 EVD outbreaks have been registered in Sub-Saharan Africa in the past four decades, causing over 34,356 infections and 14,823 deaths [8].

On the 20th of September 2022, Uganda’s Ministry of Health, together with the World Health Organization, declared yet again another Ebola outbreak of the SUDV strain in Mubende district, Central Uganda. The declaration was followed by extensive media coverage of the event. Social media sites such as Facebook and Twitter were inundated with millions of people airing and discussing their views on the outbreak. The aim of this study is to extract, analyse, and classify Ebola-related tweets published between the 20th of September 2022 and the 30th of November 2022 using deep learning sentiment analysis techniques. It is hoped that the findings of this study will provide valuable insights to the government, healthcare organizations, the general public, and other stakeholders about public sentiments towards the EVD outbreak. By understanding the public sentiment about the EVD outbreak, responsible health organizations will be able to effectively tailor communication and health policy implementation strategies to reach out to the general public [9,10,11].

Sentiment analysis, also known as opinion mining, uses natural language processing and machine learning techniques to extract, analyse, and classify opinions expressed in a body of text [12]. It has a wide range of applications, including identifying customer sentiment towards products and services, analysing political opinions, and detecting public sentiment towards events such as natural disasters or epidemics. A total 50,000 tweets related to the September Ebola outbreak in Uganda were collected between the 20th of September 2022 and the 30th of November 2022. After preprocessing the tweets, in a manner described in a later section of the study, three deep learning algorithms, namely convolutional neural networks (CNNs), long short-term memory(LSTM), and Bidirectional Encoder Representations from Transformers (BERT), were trained, validated, and tested on the resulting data to perform sentiment analysis and classify the tweets as positive, negative, or neutral. The performance of the algorithms was compared based on the most popular evaluation metrics of accuracy, precision, recall, and the F-measure.

The rest of the paper is organised as follows. In Section 2, a brief review of the relevant literature is given. Section 3 discusses the data collection procedure and preprocessing performed to make them suitable as the input to the deep learning algorithms. In addition, the section presents the models used in this study. In Section 4, the study results are given. Section 5 discusses the results, while Section 6 presents the conclusions and recommendations.

## 2. Related Work

Sentiment analysis has proven to be one of the most effective techniques for extracting expressed opinions from unstructured texts [13,14,15]. Several studies have used the method to conduct epidemic/pandemic sentiment analysis as well using lexicon-based methods, machine learning, and deep learning classifiers [13,16,17,18,19]. For example, Chintalapudi et al. [16] compared the performance of a deep learning Bidirectional Encoder Representations from Transformers (BERT) model with three other machine learning models; namely logistic regression (LR), support vector machines (SVMs), and long short-term memory (LSTM) in analysing sentiments in tweets posted by India Twitter users during the COVID-19 outbreak. They reported that the BERT model outperformed the other three models at predicting sentiments, achieving an accuracy rate of 89%, compared to the accuracy rate of 75%, 74.75%, and 65% achieved by LR, SVM, and LSTM, respectively.

Furqan et al. [20] employed five supervised learning techniques, namely random forest (RF), XGBoost classifier, support vector classifier (SVC), extra trees classifier (ETC), and decision tree (DT), in addition to the deep LSTM to identify sentiments in tweets related to COVID-19, which could be used to improve the management of the pandemic. Their results showed that extra trees classifiers outperformed all other trained models with an accuracy of 93%.

Leelawat et al. [21] examined the effectiveness of three machine learning models: decision tree, random forest, and SVM in determining the sentiments and intentions of the tweets about tourism in Thailand during the COVID-19 pandemic. The support vector machine algorithm provided the best results for sentiment analysis, with a maximum accuracy of 77.4%. In the intention analysis, the random forest algorithm achieved an accuracy of 95.4%.

Imran et al. [15] used a deep learning LSTM architecture utilising pre-trained embedding models to find the correlation between the sentiments and emotions of people from different cultures during the coronavirus outbreak. Tweets from six neighbouring countries, namely the USA and Canada; Pakistan and India: and Sweden and Norway were analysed. The study found a high correlation between the polarity of tweets posted from the USA and Canada and Pakistan and India. However, despite the many cultural similarities between Sweden and Norway, the correlation between the polarity of the tweets from these two countries was surprisingly low. This study differed from similar studies investigating sentiment analysis in that it used emotion emoticons to validate and test the models.

Yin et al. [22] presented a framework for detecting topics and sentiment dynamics due to COVID-19 from a collection of 13 million tweets related to COVID-19 over a period of two weeks. They found that the positive sentiment showed a higher ratio than the negative sentiment during the study period and that different aspects of COVID-19 were constantly discussed and showed comparable sentiment polarities. A topic such as “stay safe home” was dominated by positive sentiment. Others such as “people death” were consistently showing negative sentiment. Overall, the proposed framework provided insightful findings based on the analysis of the topic-level sentiment dynamics.

Martinez et al. [23] used lexicon-based techniques on 187,349 tweets gathered from May 2020 to March 2021 to examine how COVID-19 vaccine sentiment corresponded with USA vaccine deployment. The study found that most tweets expressing positive sentiments coincided with the announcements signalling the imminent deployment of COVID-19 vaccines and that the level of positive tweets remained high at above 63% for many months thereafter.

Qin and Ronchieri [24] utilized sentiment analysis and topic modelling techniques to investigate and analyse comments related to various pandemics such as cholera, Ebola, HIV/AIDS, influenza, malaria, Spanish influenza, swine flu, tuberculosis, typhus, yellow fever, and Zika. They found that people’s discussions were primarily focused on malaria, influenza, and tuberculosis. Kaushik and Bhatia [25] proposed a framework for extracting tweets related to coronavirus all over the world and employed unsupervised learning techniques (K-means and hierarchical clustering algorithms) to gain insight into the situation in different countries. The study found that there were mixed emotions among people with a high degree of pessimism.

Odlum and Yoon [26] examined and evaluated the public sentiment on HIV/AIDS from Twitter data on World AIDS Day 2013. Song et al. [27] proposed a novel text representation model for a short-text sentiment analysis framework based on probabilistic linguistic terms and relevant theory. Their proposed framework combined both supervised learning and unsupervised learning.

In this study, we specifically adopted the implementation techniques from Chintalapudi et al. [16] for the BERT model and from Imran et al. [15] for the LSTM model, tailoring these approaches to fit the linguistic and cultural specificities of the Ugandan context. This adaptation is crucial, as it allows us to leverage robust, established methodologies while ensuring that our analysis is sensitive to the local dialect and sentiment expression. By integrating these proven techniques within our unique regional focus, our research extends the geographical and contextual scope of sentiment analysis, contributing vital insights into the interplay between public sentiment and health policy in regions heavily affected by misinformation. This contribution is not only a testament to the versatility of sentiment analysis, but also serves as a valuable model for similar studies in other under-represented areas.

## 3. Materials and Methods

**Figure 1:**
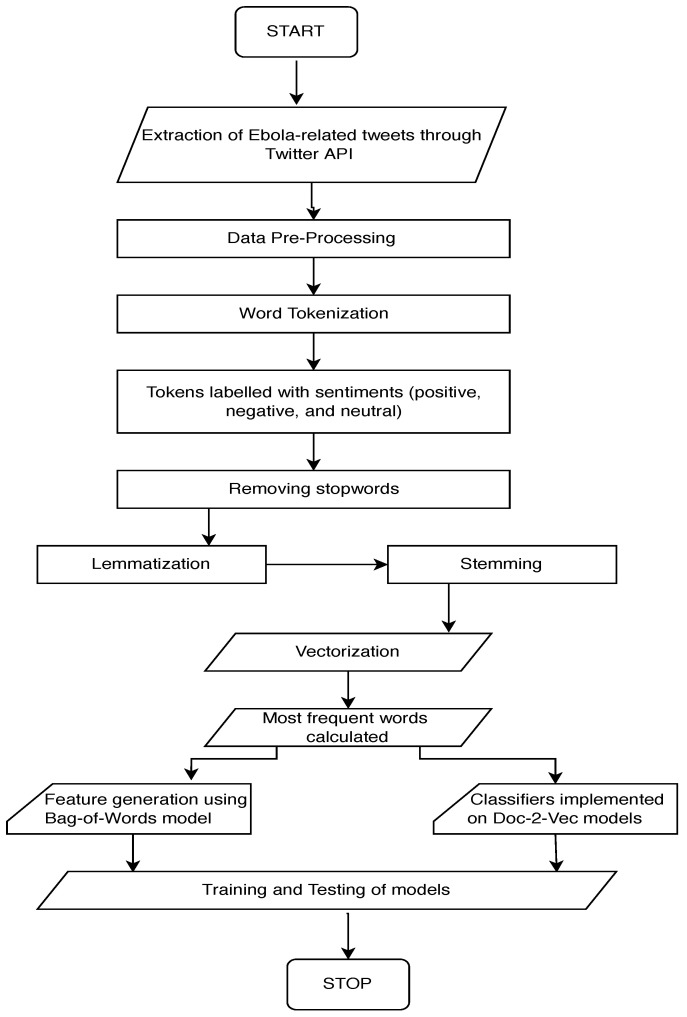
The implementation structure of the sentiment classification algorithms. Adapted from [13].

This section describes in detail the dataset, methods, and evaluation metrics that were used during the training and testing of the sentiment classification algorithms. We utilized the Python *natural language toolkit* (*NLTK*) package for most of the preprocessing tasks [28], as well as the in-built Python package “*re*” for performing regular expression operations. The classification algorithms were implemented following a series of preprocessing phases as detailed in Figure 1.

### 3.1. Dataset

**Figure 2:**
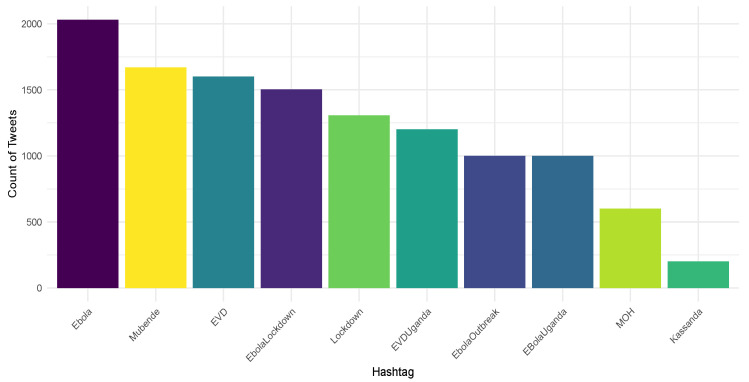
Most common hashtags in tweets during the Ugandan Ebola outbreak.

The dataset used in this study comprised over 13,629 tweets related to the Ugandan Ebola outbreak posted between the 20th of September 2022 and the 30th of November 2022. The tweets were extracted using the Twitter Search API with the Python *Tweepy* library. The extraction was performed using keywords such as “Ebola”, “Ebola virus”, “Ebola outbreak”, “EVD”, “Ebola Uganda”, and “Ebola crisis”. Only English-language tweets were included in the dataset. Figure 2 below shows the top-10 most common hashtags that emerged from this dataset, providing insights into the primary concerns and topics of discussion among Twitter users during the outbreak.

### 3.2. Data Preprocessing

Preprocessing of data is a very vital step in machine learning modelling, as it affects the accuracy of the models. In this study, tweets were cleaned to remove redundant text and symbols that are generally associated with tweets, such as: stop words (e.g., a, an, as, etc.), usernames, symbols (e.g., @, RT, #, URLs), punctuation marks, and numeric values (alpha-numeric words were not removed). The removal of these symbols and text also helps in reducing the noise in the dataset. Furthermore, duplicate tweets and tweets containing non-English words were eliminated, resulting in a reduction of the dataset from the original 13,629 tweets to 8395 tweets. Finally, to make the data uniform and reduce noise, all the tweets were converted to lowercase [20]. The other preprocessing tasks performed on the data are explained in the subsections below.

#### 3.2.1. Word Tokenization

In sentiment analysis of tweets, word tokenization is the process of breaking down the text of a tweet into individual words, also known as *tokens*. This is a very important step in the sentiment analysis process, as it allows a language model to understand the context and meaning of each word in the tweet.

#### 3.2.2. Token Labelling

**Figure 3:**
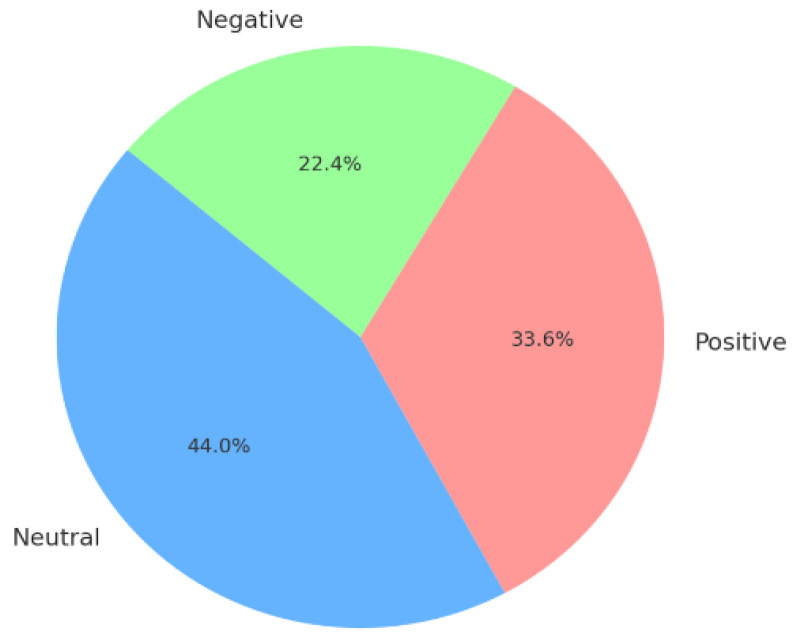
Count of the number of occurrences of each sentiment category.

After tokenization, the resulting tokens along with their labels are used as the input to a sentiment analysis model. Token labelling assigns a label or tag to each token based on its context and meaning within the tweet [29]. For this study, tokens were either assigned positive, negative, or neutral tags using the Python *NTLK* package [28]. A negative sentiment tag typically refers to tweets expressing concerns, fear, or distress related to the Ebola outbreak. A neutral tag refers to a tweet that conveys factual information, updates, or statistics about the outbreak without expressing any emotional tone, whereas a positive tag represents a tweet expressing optimism, relief, or praise for efforts towards the Ebola outbreak such as applauding healthcare worker’s successful containment measures or supportive international aid. The overall dataset sentiment distribution consisted of 1888 negative tokens, 2820 positive tokens, and 3693 neutral tokens, as illustrated in Figure 3.

#### 3.2.3. Stemming

Stemming is a preprocessing technique used in text processing to reduce inflected words to their root form [30] so that words with the same stem are recognized as the same word, even if they have different endings. For example, the words “bringing” and “brought” would be reduced to the root word or stem “bring”. This technique is very useful in natural language processing tasks such as text classification and information retrieval [31,32].

#### 3.2.4. Lemmatization

Lemmatization is a text-processing technique similar to *stemming*, but it takes into account the context and grammatical function of a word in order to produce a valid base form, known as the *lemma* [33]. Lemmatisation aims to simplify inflected words to their base form, making them more meaningful and conveying the same meaning as the original words. It helps to match synonyms using a thesaurus. For example, when one searches for “hot”, the word “warm” is matched as well.

After completing all the preprocessing steps, the Python library, *TextBlob*, for processing textual data, was applied to the data to find and assign sentiment scores.

### 3.3. Deep Learning

Deep learning is a subset of machine learning that uses neural networks with multiple layers to learn from data [34]. In recent years, deep learning-based techniques have become popular for sentiment analysis. The most widely used deep learning techniques are CNNs [35], LSTM [36,37], and Bidirectional Encoder Representations from Transformers (BERT) [38,39,40]. The following subsections provide a brief general description of the CNN, LSTM, and BERT models as applied to sentiment analysis.

#### 3.3.1. Convolutional Neural Network

Convolutional neural networks (CNNs) are one of the deep learning models used for sentiment analysis in this study. CNNs are a type of deep learning model that has been proven to be effective for text classification tasks [41,42,43]. They are composed of multiple layers of neurons, with each layer performing a different operation on the input data.

The first layer of a sentiment analysis CNN model is the embedding layer, which maps each word in the input text to a dense vector representation. This allows the model to capture the semantic meaning of the words and their relationships to each other [35]. The output of the embedding layer is passed through a series of convolutional and pooling layers, which extract features from the input text. Finally, the output of the convolutional and pooling layers is passed through a fully connected layer, which performs the actual classification. We based our implementation on the architecture proposed by [44], but with slight modifications and adjusted hyperparameters.

#### 3.3.2. Long Short-Term Memory

Long Short-Term Memory (LSTM) is a type of Recurrent Neural Network (RNN) that is commonly used for sentiment analysis. LSTMs are designed to handle sequential data, such as text, by learning to remember information for longer periods of time [45]. This allows them to understand the context of a sentence or a whole paragraph, which is important for determining the sentiment of the text. LSTMs are trained on large datasets of labelled text, where the sentiment of each text is labelled as positive, negative, or neutral. The model learns to identify patterns and features that are indicative of a particular sentiment by adjusting the weights of the network through backpropagation.

Compared to CNNs, LSTM models are a bit more complicated to interpret due to their complex structure [46]. In addition, LSTM models require large amounts of labelled data and computational resources to train.

#### 3.3.3. Bidirectional Encoder Representations from Transformers

Bidirectional Encoder Representations from Transformers (BERT) is a deep learning model that has been used in several natural language-processing tasks, including sentiment analysis [38,39,40]. BERT is a Transformer-based model that uses an attention mechanism to learn the context of a sentence by attending to different parts of the input text [47]. In a sentiment analysis task similar to LSTM, BERT is trained on large datasets of labelled text. The model learns to identify patterns and features that are indicative of a particular sentiment by adjusting the weights of the network through backpropagation.

One of the key advantages of BERT is that it can be fine-tuned on a smaller labelled dataset, which is helpful when working with limited data [48]. However, BERT requires a considerable amount of computational resources to train, and it may not be suitable for small-scale or low-resource devices.

### 3.4. Evaluation Parameters

We evaluated the classification performance of different algorithms using the most common evaluation metrics of accuracy, precision, recall, and the F-measure.

#### 3.4.1. Accuracy

Accuracy is a performance evaluation indicator that measures the percentage of correct predictions made by the model. It is mathematically defined as:

(1) $$Accuracy=\frac{TP+TN}{TP+TN+FP+FN}$$

where

True Positives (TP) —the number of correct positive predictions.
True Negatives (TN)—the number of correct negative predictions.
False Positives (FP)—the number of incorrect positive predictions.
False Negatives (FN)—the number of incorrect negative predictions.

#### 3.4.2. Precision

Precision is the measure of the proportion of true positive predictions among all the positive predictions made by the model. It is calculated as:

(2) $$Precision=\frac{TP}{TP+FP}$$

#### 3.4.3. Recall

Recall indicates the proportion of true positive predictions among all the actual positive examples in the dataset, and it is calculated as:

(3) $$Recall=\frac{TP}{FP+FN}$$

#### 3.4.4. F1 Score

The F1-Score is the harmonic mean of precision and recall, which gives equal weight to both precision and recall. It is calculated as:

(4) $$F1\;Score=2*\frac{Precision\times Recall}{Precision+Recall}$$

### 3.5. Modelling

This study investigates and compares the performance of three deep learning algorithms, CNN, LSTM, and BERT, on the task of deriving sentiments from tweets. The dataset utilized in this study consisted of over 8395 distinctive tweets. To prevent the algorithms from overfitting, the dataset was divided into three sets: the training set and the testing set in a 4:1 ratio. The training set was subsequently partitioned into a validation set, which constituted 20% of the training data, and was used, first, to fine-tune the hyperparameters of the algorithms and, second, to evaluate the performance of the algorithms throughout the training process. A range of parameters was used to train and refine the three models—iterating through multiple rounds of training and fine-tuning until satisfactory results were achieved. The implementation details of each algorithm are discussed in the next three subsections.

#### 3.5.1. CNN Model

The CNN model was built using six convolutional layers. Two of these layers had 32 filters, two with 64 filters, and the last two with 128 and 256 filters, respectively. Additionally, the model included a GlobalMaxPooling1D layer and a flattened layer. Dropout layers with a rate of 0.5 were incorporated into the model to ensure regularisation. These layers were subsequently connected through two dense layers to perform sentiment classification. The selection of these specific parameters was the outcome of extensive experimentation and fine-tuning practices. Each configuration was methodically tested to determine its impact on model performance, ensuring that the final parameters provided the most optimal results for sentiment classification. For a comprehensive view of the parameters used during the training of the CNN model, please refer to Table 1.

#### 3.5.2. LSTM Model

**Figure 4:**
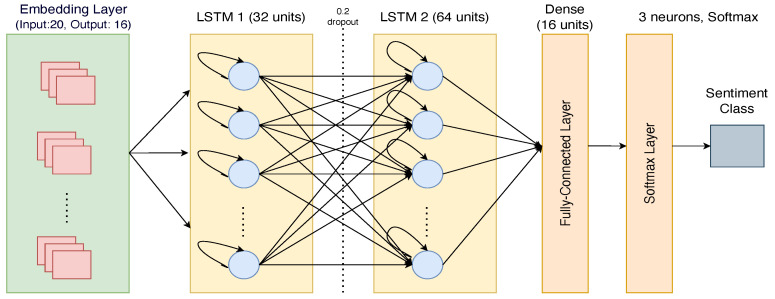
Sequential long short-term memory (LSTM) hyperparameters and architecture for sentiment analysis.

The LSTM model architecture used in this study consisted of four main layers—an Embedding layer, two LSTM layers, one fully connected layer, and a final output layer. The Embedding layer took input data of size 20, with 100 unique words in the vocabulary, and output a lower-dimensional representation of size 16 for each word. The two LSTM layers had 32 and 64 neurons, respectively, with a dropout rate of 0.2 to prevent overfitting during training. The fully connected layer had 16 units, which acted as an intermediary layer between the LSTM layers and the final output layer. The final output layer consisted of three neurons responsible for classifying the sentiment of the input text using a softmax activation function. The configuration details of the LSTM hyperparameters and the overall architecture are shown in Figure 4.

The selection of the parameters for the LSTM model was an iterative process that involved training and evaluating the model’s performance under various conditions. After several rounds of experimentation and fine-tuning, the final set of parameters was selected. The model was trained for 10 epochs, indicating the number of times the entire training set was processed. To balance the computational efficiency and model accuracy, a batch size of 64 was used. The Adam optimizer was chosen to manage the update of the model weights, as it has been shown to be effective in optimizing deep learning models [49,50]. Additionally, a learning rate of 0.0001 was set to control the step size when performing the update, as it affects the convergence speed of the model during training.

#### 3.5.3. BERT Model

We developed a BERT-based sentiment analysis model employing the “bert-base-uncased” pre-trained BERT model [47], which consists of 12 transformer layers and contains approximately 110 million parameters. We utilized the corresponding tokenizer from the *Hugging Face Transformers* library in Python for preprocessing the textual data. The architecture of our BERT model involved an embedding layer obtained directly from the pre-trained BERT, which effectively captures contextual relationships within the text. Following the embedding layer, the architecture includes two bidirectional LSTM layers, each with 32 units and “return_sequences = True”. This configuration allows the model to maintain sequence information across the embeddings, which is crucial for understanding the context within text data.

To transform the variable-length output of the LSTMs into a fixed-length representation suitable for classification, we applied a GlobalMaxPooling1D layer. This layer extracts the most significant features from the sequence of LSTM outputs, which are then fed into the subsequent layers of the network. For regularization, we incorporated dropout layers with a dropout rate of 0.5 after each LSTM and before the final classification layers. This approach helps in reducing overfitting by randomly deactivating a fraction of the neurons during the training phase.

**Figure 5:**
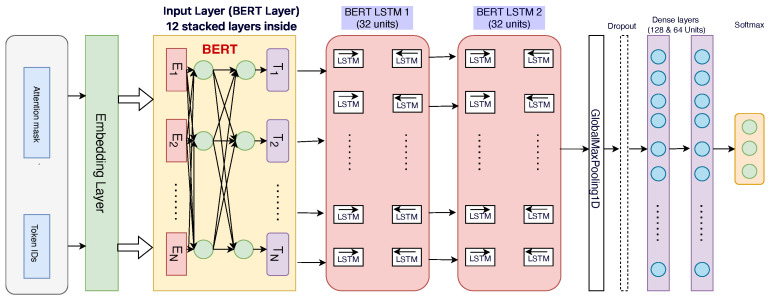
The BERT model hyperparameters and the overall architecture.

The model further includes two dense layers with 128 and 64 units, respectively, both utilizing the ReLU activation function to introduce non-linear transformations. These layers are important for learning complex patterns in the data. We conducted the training over 30 epochs using the Adam optimizer, with sparse categorical cross-entropy as the loss function, suitable for multi-class classification tasks. The BERT layers were kept frozen during training to preserve the pre-trained weights, a strategy proven to enhance performance while limiting the number of trainable parameters [51]. Finally, the training was optimized by setting a batch size of 64 and incorporating early stopping based on validation performance to prevent overfitting and shorten the training time without compromising model accuracy. The overall architecture is shown in the Figure 5.

#### 3.5.4. Word Cloud

**Figure 6:**
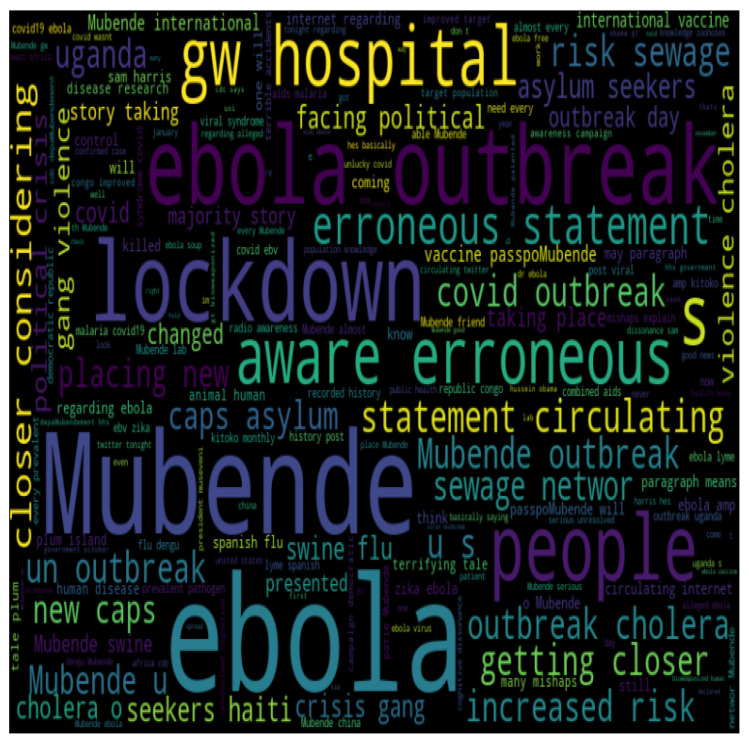
Word cloud representation of the most frequent words found in tweets about the Ebola outbreak, highlighting the key terms and topics associated with the crisis. The size of each word reflects its frequency in the text corpus.

According to Figure 6, the most frequently used words in the tweets were *ebola, lockdown, Mubende, outbreak, hospital, people, and erroneous*.

## 4. Results

**Figure 7:**
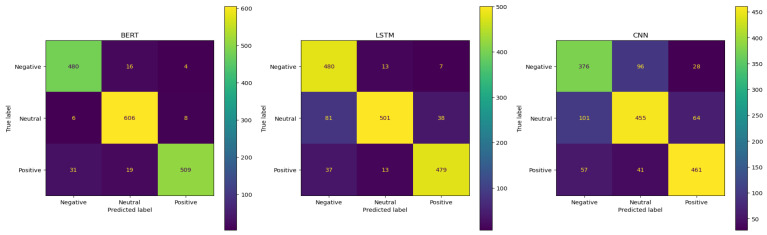
Confusion matrices for BERT, LSTM, and CNN models, illustrating the comparative performance of each model.

In this study, we tested and evaluated the performance of three trained deep learning models at predicting sentiment from Twitter data related to the Ebola outbreak in Uganda in 2022. The results in Table 2 indicate that the BERT model exhibited superior performance in classifying sentiments as neutral, positive, or negative to the CNN and LSTM models. Thus, the BERT model accurately classified 480 tweets as negative, 606 tweets as neutral, and 509 as positive out of 500, 620, and 559 tweets, respectively. Meanwhile, the CNN model obtained accurate predictions for 376 out of 500 negative sentiments, 461 out of 559 positive sentiments, and 455 out of 620 neutral sentiments, whilst the LSTM model achieved correct classification for 501 neutral sentiments, 479 positive sentiments, and 480 negative sentiments out of 620, 559, and 500, respectively. See the confusion matrices in Figure 7 for more details.

The results of the evaluation are presented in Table 2.

## 5. Discussion

The results emphasize the effectiveness of employing Transformer-based architectures, such as the BERT model, in tasks related to natural language processing, such as sentiment analysis. This indicates a transition towards more advanced deep learning techniques that have the capability to capture semantic relationships within text data comprehensively. Consequently, it opens up new possibilities for sentiment analysis, sentiment-aware content curation, and opinion mining on social media platforms.

Furthermore, the study reveals that a significant proportion (44.0%) of the tweets carried a neutral tone, signifying that most Twitter users shared factual information, updates, or statistics about the Ebola outbreak without conveying any emotional bias. Meanwhile, 33.6% of the tweets conveyed positive sentiments: reflecting the optimism, relief, or praise directed towards the country’s efforts in responding to the Ebola outbreak, and 22.5% of tweets conveyed negative sentiments in which individuals expressed fear or distress.

## 6. Conclusions

In this study, the comparative performance of three deep learning models, CNN, LSTM, and BERT, at carrying out sentiment analysis on tweeter data related to the 2022 Ebola outbreak in Uganda was investigated. The study found that the BERT model is superior to the other two models at performing sentiment analysis on several metrics, including accuracy, precision, and recall.

The success achieved by the BERT model in comparison to conventional deep learning models emphasizes the importance of continuing to explore and integrate these cutting-edge deep learning methodologies. Such efforts are crucial to fully harness the potential of sentiment analysis in comprehending human emotions and opinions as expressed in online conversations, such as those on Twitter. These advancements will undoubtedly prove invaluable to health experts and other stakeholders, empowering them to make well-informed decisions during outbreaks like Ebola and COVID-19.

Considering these findings, it is also evident that communication strategies should incorporate sentiment analysis of social media data for future outbreaks. This approach promises to enhance the effectiveness of communication efforts by tailoring them more precisely to the prevailing sentiments within the online discourse.

## Figures and Tables

**Table 1:** Parameters used to train the CNN model.

Parameter	Value
Learning Rate	$1\times 10^{-4}$
Epochs	10
Batch Size	64
Kernel Size	3×3
Dropout	0.5
Activation	Softmax
Optimizer	Adam
Loss	sparse_categorical_crossentropy

**Table 2:** Results achieved by different models.

Models	Accuracy	Precision	Recall	F1-Score
CNN	0.77	0.81	0.74	0.74
LSTM	0.87	0.91	0.84	0.85
BERT	0.95	0.96	0.93	0.94

## Data Availability

The dataset and code used for Twitter data scraping, training, validation, and testing of algorithms are accessible through the following GitHub link: https://github.com/mirugwe1/Sentiment_Analysis.

## Abbreviations

The following abbreviations are used in this manuscript:

EVD	Ebola virus disease
CNN	convolutional neural network
LSTM	long short-term memory model
BERT	Bidirectional Encoder Representations from Transformers
